# Glasgow Royal Infirmary

**Published:** 1829-04-01

**Authors:** 


### GLASGOW ROYAL INFIRMARY.

### SYNOVITIS.

Traumatic depositions of lymph and pus in various parts and structures of the body are at present attracting much attention, not only in this country, but also abroad. We have already given several articles on the subject, and flatter ourselves, "for pride attends us still," that we may have been the means of directing to its investigation the minds of our surgical brethren, and thereby eliciting valuable information. When the able and elaborate paper of Mr. Arnott shall be published in the Medico-Chirurgical Transactions, we purpose very fully considering the question, and therefore defer till that period any general remarks we may venture to make. In order, however, to conclude our notice of the surgical report of Dr. Couper from the Glasgow Infirmary, we shall briefly narrate two cases of what he has denominated, not very happily in our opinion, "synovitis."

CASE 1. *Excision of Part of the Tunica Vaginalis—Formation of Matter in the Left Knee and Right Shoulder.*

A sailor, fifty-six years of age, was admitted on the 26th December last, with a moderate sized hydrocele on the right side, which was treated on the following day by evacuating the fluid with a lancet, and excising a minute portion of the tunica vaginalis. On the 12th of January the patient was dismissed cured, but returned to the Infirmary three days afterwards with a small collection of pus under the integuments at the site of the wound. The part was laid open and dressed, and by the 27th of January the wound had almost cicatrized, but whilst placing some leeches on another individual that day, he experienced a rigor. Next day he complained of occasional obtuse pain in the calf of the left leg and left knee, but this shifted in the evening to the opposite limb. On the 29th, the pains were complained of in both legs alternately, and after a rigor that evening the right shoulder was also attacked. On the 30th, the pain was most severe in the latter situation, but in no one place could swelling or discolouration be discovered. His pulse was now quick, his tongue brownish, and he complained of oppression at the præcordia. In the evening of the 30th, he was seized with another violent rigor, hiccuped occasionally, and complained much of pain in the right shoulder, left knee, and left calf, which last was now very tense and swollen, without discolouration. He sank on the morning of the 31st, "*notwithstanding*" the free use of purgatives, antimonials, leeches, and evaporating lotions from the onset.

*Dissection.* The "integuments" of the calves of both legs, especially the left, were distended with brownish serum, and the cellular connexions of the muscles also contained a similar fluid. A few drachms of thick pus were found in the left knee-joint, *without vascularity or swelling of the lining membrane.* The integuments on the inner side of the right shoulder were similarly affected; there was a small quantity of thick pus in the bursa under the deltoid muscle, and a very small quantity within the joint; the thoracic and abdominal viscera were healthy.

CASE II. *Retention of Urine relieved by the Bougie—Purulent Depôts in the Left Knee and Elbow-joint.*

James M'Cormick, a porter, ætatis 43, was admitted Oct. 13th, 1827, with retention of urine from chronic enlargement of the prostate gland. With some difficulty a good sized elastic catheter was passed into the bladder; and on the 17th inst., four days after admission, he had recovered the power of voiding his urine. On that day he complained of pains in both shoulders, for which vinum Colchici was prescribed. After two days the pains removed to the left knee and left elbow; they were very severe, and accompanied with swelling in both joints. On the 24th, some external redness was perceptible, and on the 27th, fluctuation being distinct near the elbow, a puncture was made, and about an ounce of pus discharged. The affection of the left knee followed the same course. On November 2d, although the patient was evidently sinking, the abscess was opened, and 8 ounces of pus evacuated. On the following night he died. The fever in this case was considerable, and during the last seven days of the disease, was accompanied with delirium, and a deep yellow colour of the whole skin. Leeches, purgatives, opiates, and cataplasms, were employed freely with very partial advantage.

*Dissection.* The left elbow-joint contained much thick pus, communicating with the external opening by a sinus behind the external condyle, which, within the joint, was denuded of cartilage and rough, as was likewise the head of the radius. The left knee-joint was filled with pus, which also passed up to the extent of five inches betwixt the vastus externus and the femur. The prostate was double its natural size, and a false passage was found to be connected with the urethra. The viscera of the thorax and abdomen were natural.

"Every hospital surgeon is well aware, that, besides cutaneous erysipelas, the inmates of large hospitals are also liable to certain insidious, acute inflammatory affections, supervening in patients admitted for other disorders, especially in those who have undergone any surgical operation, and in general proceeding rapidly, notwithstanding the most active treatment, to a fatal termination. In such cases the disease is usually seated in the serous linings of the thorax and abdomen; but why may not the synovial membranes also be liable to analogous attacks? Acute synovitis is, in general, distinctly traceable to partial application of cold, or to mechanical injury of the joint. In neither of the cases now reported, however, did any such local cause exist; besides, the disease did not appear in one, but simultaneously in several different and distant parts of the synovial system."

Dr. Couper remarks that such insidious attacks are usually seated in the *serous* linings of the chest or the abdomen, in which our experience differs widely from his. The most common seat of these insidious inflammations, as far as we can judge, is either in the hepatic or pulmonary structure, and *not* in the cavities lined by serous membrane. Next in point of frequency to that of the liver or the lungs, we should place the affection of the pleura, then the depôts of matter in the joints, and, last of all, peritoneal inflammation. Dr. Couper has allowed much confusion to creep into his argument, short though it be; for he evidently confounds these deposits of pus in the joints with acute inflammation of the synovial membrane, or, as he himself terms it, acute synovitis. The fact, however, is, that the causes, the symptoms, the termination, and the appearances *post mortem*, are totally different, and, therefore, it cannot be adviseable to lump such opposite affections together. Dr. Couper will, also, discover, if he meets with such cases as have lately occurred in some of the hospitals of this metropolis, that depletion is totally ineffectual in arresting the disease, be it carried to what extent it may.

---

### III.

### NEW YORK HOSPITAL.

### On the Mercurial Treatment of Fever. By Dr. Brown.

### XXXV.

### GLASGOW ROYAL INFIRMARY.

"SINGULAR CASE OF EMPHYSEMA."*

A feeble, emaciated old man of 77, was admitted into the Royal Infirmary of Glasgow, on the 2d of December last, labouring under dysuria and enlargement of the prostate gland. The patient was also affected with cough and dyspnœa, his appetite was gone, and he seemed fast hastening to his grave. Occasional laxatives, generous diet, and a liberal allowance of wine kept him alive for a fortnight or so, when suddenly emphysema "of the common integuments" appeared about the root of the neck and on the chest, and rapidly extended to the other parts of the body. "It was ascertained that he had received no injury, and it was therefore concluded that the emphysema arose from interlobular emphysema of the lung, extending itself upwards, through the cellular tissue of the mediastinum, and the rest of the neck." Scarifications were employed, but in three or four days the patient died.

*Sectio Cadaveris.* The cellular tissue of the face, neck, thorax, and abdomen was much distended with air. On removing the integuments of the thorax, the air was at once seen to have issued from an opening in the right side of that cavity, formed by a disjunction of the connexion between the cartilage of the third rib and the body of that bone. That this disjunction was not altogether of recent formation was evident, from the smooth and polished appearance of the sides of the opening formed by the muscular tissue connecting the lateral parts of the rib and cartilage. On removing the sternum and cartilages, an opening was seen in the substance of the lung,

---

* *Med. Gaz. Jan.* 31. 1829.

continuous with the opening in the cavity of the thorax. This opening conducted into a cavity about the size of a walnut, containing no purulent secretion. Around its edges the lung *adhered very firmly to the parietes of the thorax, and several other adhesions connected the remaining part of the lung to the chest.* Several other cavities were found in the substance of this lung, and also in the upper portion of the opposite lung. The prostate was found considerably enlarged, and an abscess at the inferior part of the bladder.

The above dissection which we have copied verbatim, from the report in our contemporary, though not very lucidly detailed, is yet one of interest. The case was evidently one of pneumo, we beg pardon, pneum*a*-thorax, though the air was not extravasated into the general cavity of the pleura, in consequence of the adhesions of the lung to the side of the chest, more especially immediately around the aperture. The manner in which the cartilage and body of the rib became disjoined, and allowed of the emphysematous escape of the air, is obscure; indeed we can form no plausible conjecture to account for it. In such a case it is obvious that no operation is required, though perhaps compression applied to the opening between the body of the rib and its cartilage would have proved effectual in arresting the egress of the air. The nature of the case, however, was not in the least degree, it seems, suspected during the patient's existence, and even if it had been made out, the age of the patient and other circumstances would have rendered any chance of recovery desperate.

---

### XXXVI.

### LXIII.

### GLASGOW ROYAL INFIRMARY.*

We think we may with pardonable vanity congratulate ourselves on having been the means of encouraging our hospital surgeons and physicians to publish many useful and valuable reports. Would to heaven that the hint were more generally acted on, and that every provincial hospital and infirmary had medical officers able and willing to sacrifice some trifling time and trouble with the noble aim of conferring information on their brethren, and almost inestimable benefit on science. That march of intellect, which, however scoffed at by those in the rear, is daily advancing with giant strides, and nowhere more rapidly than in our own profession, will rouse, we trust, to a sense of shame, if not to one of duty, the indolent portion of our "public functionaries." We say a portion, for many, we are happy to say, have not shewn that apathy which seems to have sealed with a leaden weight the slumbers of the rest. Amongst the most active may be ranked the surgeons of the Glasgow Infirmary, from whom have emanated some of our most interesting hospital reports; of the former we have given very ample accounts, and we hail the present from Dr. Maclachlan as a worthy successor of those which have preceded it.

I. Compound Fracture of the Cranium after Symptoms of Compression—Trephine applied on the 8th Day.

D. M'Leod, 11 years of age, was employed, on the 4th of last August, rivetting a boiler, when one of the rivets was driven with considerable force into his head. Hæmorrhage to some amount took place from the wound, and two hours afterwards the boy was admitted into the

* Report of cases treated in the surgical wards. By G. M. Maclachlan, M.D. Glasg. Journ. No. V.

infirmary with a wound an inch and a half in length over the occipital angle of the right parietal bone, through which a fracture of that bone was discovered depressed full half an inch, and having a diameter of nearly an inch in every direction. The wound bled freely; there was slight stupor, but the patient answered questions distinctly; the pupils were natural; the pulse 84; respiration easy and natural. The wound was brought together and lightly dressed—a scruple of calomel—laxative enema. He passed a quiet night, and had one stool, but the pulse next morning had risen to 110, and he was therefore bled to ten ounces. At Dr. Maclachlan's visit, the intellect was perfect, he did not complain of much head-ache, the pupils were natural, but he fancied that objects at a distance were near him, the pulse was 120, and he suf-suffered from occasional retching. The bleeding was repeated, with cold applications to the head, and a laxative enema in the evening. By the 8th, the pulse had subsided to 78, and the head-ache had left him; but, on the 10th, there was some increase of pain in the site of the fracture, and the pulse was 80. On the 11th, the report was, that he had dozed almost constantly since the preceding afternoon, had vomited his breakfast that morning, answered questions distinctly but with much reluctance, and lay in an apparently exhausted state, with the pupils dilated and sluggish, the pulse 65, the respiration natural.

Under these circumstances, it was determined in consultation to operate. On laying bare the bone, an opening, nearly an inch in diameter, was found, the broken fragments being depressed nearly at right angles with the sound bone, and consequently having their points pressing directly inwards on the brain. Two applications of the trephine were required, when the brain resumed its proper level, and the hitherto weak pulsations became strong. The dura mater was slightly wounded at several points by the spiculæ, and minute portions of brain were seen floating off with the blood. The boy immediately became more lively, the pupils active, and pulse got up from 65 to 96. The scalp was laid down, and retained by two stitches, and the parts lightly dressed. He went on well till the morning of the 19th, when his expression was dull; he complained of uneasiness in the site of the wound, the discharge from which was rather diminished; the pupils were sluggish; the pulse 102. He was bled to eight ounces, and a laxative enema given, after which no bad symptom appeared. The wounds healed, and the boy was discharged cured on the 6th of September.

Dr. Maclachlan observes that, "it is now, I believe, generally conceded among surgeons, that when a fracture of the cranium with depression has taken place, unless symptoms of compression or cerebral irritation be present, the case should be left to Nature; removing only, where there is a wound of the scalp, such fragments of bone as may be readily extracted."

We cannot allow that this position is so generally conceded amongst surgeons as Dr. Maclachlan would have us believe; in fact, we *know*, from their published sentiments, that not only is it doubted, but actually denied by the two most eminent in this country at the present day, we allude to Sir Astley Cooper and Mr. Brodie. Both these gentlemen agree in drawing a distinction between simple and compound fracture of the cranium, as well as in establishing a material difference in point of treatment. In compound fracture with depression Sir Astley operates, whether there be symptoms or whether there be not, and Mr. Brodie's words are these:—"In those cases in which a depression of bone exists, *without* any symptoms, or *with only* trifling symptoms arising from it, the surgeon can follow no better general rule than this: if the depression be exposed in consequence of a wound of the scalp, let him apply the trephine and elevate the depression; but if there is a depression without a wound of the scalp, in consequence of the accident, let him not make such a wound by an operation." Under these circumstances we conceive that Dr. M. was somewhat unguarded in his remark, and if we might be permitted to "hint a fault" in practice, we should say that his own case proved his error. The case was one of compound fracture with depression; the bone was not raised, and what was the consequence? Secondary symptoms, for which the trephine was required after all, and required, too, at a time when, cæteris paribus, the chances of success are infinitely lessened. The patient recovered, but had this same accident happened to an adult instead of to a boy, we would not have answered for similar good fortune. The case, in fact, is one of many strongly in favour of Sir Astley Cooper's position, and we earnestly recommend our surgical brethren to pay the question that consideration it imperiously, in our opinion, demands.

### II. Taliacotian Operation performed with Success.

Since the supplemental noses which, the poet tells us, "Taliacotius from the brawny part of porter's b—m first cut," many such useful appendages to the human face divine, have since been fashioned by the barber-surgeons, and surgeons not barbers, who have followed them. The following, we are told, was moulded and applied rather for use than for show.

Ann Nicholson, æt. 37, was admitted on the 16th Aug. 1827, with no part of the nose remaining, except the right ala and the columna. All had been swept away, leaving an unsightly chasm, bounded at the sides by the nasal processes of the superior maxillary bone, and above by the os frontis. As the edges of the opening were covered, in most parts, only by a red, shining cuticle, which the slightest injury caused again to ulcerate, it was thought that, by giving them a soft covering from the neighbouring parts, a recurrence of these ulcerations might not only be prevented, but the hideous deformity in some degree remedied.

"The case was by no means a promising one, but the patient being willing, and a consultation having sanctioned it, the operation was performed in the following manner: The right ala and columna being retained, and the edges of the chasm being pared all round, a flap, two and a half inches in length, and one and a half in breadth, having somewhat the shape of a jibsail, was taken from the left side of the forehead, the integuments over the centre having been destroyed by previous ulcerations, and now occupied by linear cicatrices. The flap was left attached at its lower and narrower part, near the inner angle of the orbit; a half turn was given to it at this point; it was then laid down and fitted to the raw edges of the opening, and retained *in situ* by four stitches. A piece of India rubber was introduced by the new-made nostril, and passed upwards so as to raise the flap in imitation of the bridge of the nose. Soft lint was laid along the raw edges, and over all a light compress. The wound of the forehead was approximated, as much as possible, by adhesive straps. The dressings were removed on the fourth day, when the flap was found in perfect apposition, and united throughout most of its extent. Thereafter it was dressed daily, and in about three weeks had quite cicatrized, remedying very materially the deformity, and leaving a soft cushion over the sharp edges of the nasal processes of the superior maxillary bones. The cicatrix on the forehead became of very small dimensions—a mere line. She continued in the house till 6th November, when she was discharged, in good health, and greatly amended in appearance. It should be mentioned, that the communicating part of the flap never was divided, the prominence formed by the twist serving to remedy the deformity arising from the want of the bones of the nose."

### III. Fungous Tumours from the Gum and Alveoli.

Most surgeons have had opportunities of witnessing the hard, immoveable, red, and, for the most part, exceedingly vascular, tumours which grow from the gum, generally from that of the upper jaw. The structure of these tumours is frequently exceedingly like scirrhus, at least it is semicartilaginous, and, in one or two instances, we have found on the section a portion of sabulous matter or bone. Three cases of the kind are related by Dr. Maclachlan. In the first, the tumour occupied the situation of the two middle incisors of the lower jaw, and had originated two years previously, in consequence of breaking one of them. The disease was removed by the knife, but the profuse hæmorrhage which ensued required the repeated application of the actual cautery, together with compression, by means of a firm roll of lint, and a bandage passed across the angles of the mouth and round the chin. The wound healed kindly, and no appearance of reproduction of the disease has taken place.

In the second instance, the tumour, which was firmer, more nodulated, and paler in colour, was of twenty years' standing, had already been twice removed, and grew from the upper jaw. It was extirpated by the knife, and the actual cautery afterwards applied. The third was one of a similar history and structure, apparently rather a production of the bone, than of the covering soft parts, removed by Dr. Anderson from the posterior part of the palate.

We believe it is now very generally admitted, that the mere excision of this tumour is not sufficient to ensure a cure. The actual cautery should be afterwards freely applied to the bone from which it grows, or, under particular circumstances, the saw. With all these precautions, the evil is not always completely destroyed.

IV. HOSPITAL GANGRENE.

This disease has appeared only twice; in a case of extensive ulcer on the calf of the leg, and in one of ulcer of the scrotum. The following application was speedily successful, allaying, at the same time, the constitutional disorder, by means of calomel, antimony, and opium.

"The ulcer being thickly covered with powder of Peruvian bark, spirit of turpentine was dropped on the powder, which readily imbibed it, until they formed a thick cake, then a pledgit of resinous dressing over all; after the se-second or third dressing, the ulcer lost its dirty ash-grey colour and its peculiar fœtor, its everted edges sloughed off, and a clean and healthy sore was left."

If we recollect aright, this was the practice, or nearly the practice, recommended by M. Dussassoy in the last century. We never saw it tried; but, in cases of violent sloughing, which bear a very close analogy to what is called hospital gangrene, we can strongly recommend the Peruvian or the Friar's balsam. The former we have not seen employed, but a very high character indeed has been given it, and it obviously must act like the latter, of which, from experience, we can speak in the warmest terms. The compound tincture of benzoin, or friar's balsam, is a favourite application of Mr. Brodie's to sloughing sores and stumps. In a case of the latter description, under Mr. B.'s care, the effect of the tincture, freely applied, was perfectly astonishing. Our limits compel us to stop for the present; but justice to Dr Maclachlan's excellent report, as well as to our own readers, will ensure our full consideration of the remainder.

